# Morphological and Structural Study of a Novel Porous Nurse’s A Ceramic with Osteoconductive Properties for Tissue Engineering

**DOI:** 10.3390/ma9060474

**Published:** 2016-06-15

**Authors:** Ruben Rabadan-Ros, Pablo A. Velásquez, Luis Meseguer-Olmo, Piedad N. De Aza

**Affiliations:** 1 Grupo de Investigación en Regeneración y Reparación de Tejidos, UCAM—Universidad Católica San Antonio de Murcia, Guadalupe, Murcia 30107, Spain; rubenrabadanros@gmail.com; 2 Instituto de Bioingeniería, Universidad Miguel Hernández Avda, Universidad s/n, Elche, Alicante 03202, Spain; pavelasquez@umh.es; 3 Service of Orthopaedic at Arrixaca University Hospital, UCAM—Catholic University of Murcia, Murcia 30120, Spain; lmeseguer.doc@gmail.com

**Keywords:** bioceramics, calcium silicophosphate, scanning electron microscopy, µ-Raman spectroscopy, bone response, biocompatibility

## Abstract

The characterization process of a new porous Nurse’s A ceramic and the physico chemical nature of the remodeled interface between the implant and the surrounding bone were studied after *in vivo* implantation. Scaffolds were prepared by a solid-state reaction and implanted in New Zealand rabbits. Animals were sacrificed on days 15, 30, and 60. The porous biomaterial displayed biocompatible, bioresorbable, and osteoconductive capacity. The degradation processes of implants also encouraged osseous tissue ingrowths into the material’s pores, and drastically changed the macro- and microstructure of the implants. After 60 healing days, the resorption rates were 52.62% ± 1.12% for the ceramic and 47.38% ± 1.24% for the residual biomaterial. The elemental analysis showed a gradual diffusion of the Ca and Si ions from the materials into the newly forming bone during the biomaterial’s resorption process. The energy dispersive spectroscopy (EDS) analysis of the residual ceramic revealed some particle categories with different mean Ca/P ratios according to size, and indicated various resorption process stages. Since osteoconductive capacity was indicated for this material and bone ingrowth was possible, it could be applied to progressively substitute an implant.

## 1. Introduction

Nowadays, the demand for replacement materials to fill defects is growing, especially in natural bone tissues. Implants made of synthetic polymers, ceramics, and metals are normally used for tissue repair [1,2], but none of these materials can match the quality of the original tissue they replace. In recent decades, several types of synthetic bone graft substitutes and tissue-engineered hybrids have been developed to take over the role of natural bone grafts [3,4].

Bioceramic has attracted much attention because it is low-cost, easy to produce, and offers good biocompatibility. Bone tissue mineral is a calcium-phosphate (CaP)-based apatite phase. For this reason, CaP ceramics—typically hydroxyapatite (HA) and tricalcium phosphate (TCP)—are widely used for bone tissue replacement in maxillogacial surgery and for filling defects [5,6,7]. 

Initial bone defect regeneration is favored by the presence of calcium phosphate in the bone replacement material. Despite interest having been shown in Si- and Mg-containing ceramics to develop bone implant materials [8,9,10], some research has indicated silicon to be fundamental in skeletal development [11]. 

Therefore, materials that contain silicon, calcium, and phosphorus are excellent candidates for preparing biomaterials with improved osteogenic properties. Complicated synthesis processes have been followed to acquire Si-CaP biomaterials. It is well-known that preparing single phases of crystallized Si-HA and Si-TCP materials is no easy task [12,13,14,15], which means that the mechanism of silicon-containing and/or silicon-substituted calcium phosphates is no trivial matter [16]. 

In a previous work, a simple low-cost two-step solid-state reaction process was established for obtaining a new porous Si-Ca-P-single phase ceramic called “*Nurse’s A*” in the subsystem Nurse’s A-phase—silcocarnotite within dicalcium silicate (Ca_2_SiO_4_)/tricalcium phosphate (Ca_3_(PO_4_)_2_) system [17,18,19]. 

The main purpose of the present research was to study the physical, chemical, and biological changes that take place at the implant–bone interface that controls the mechanism of direct bone tissue bonding with the implant *in vivo* by analytical scanning electron microscopy. The microstructure effect (porosity, grain size, and phase composition) on the new ceramic’s *in vivo* behavior was also studied. 

## 2. Results

### 2.1. Biomaterial Characterization

**Figure 1:**
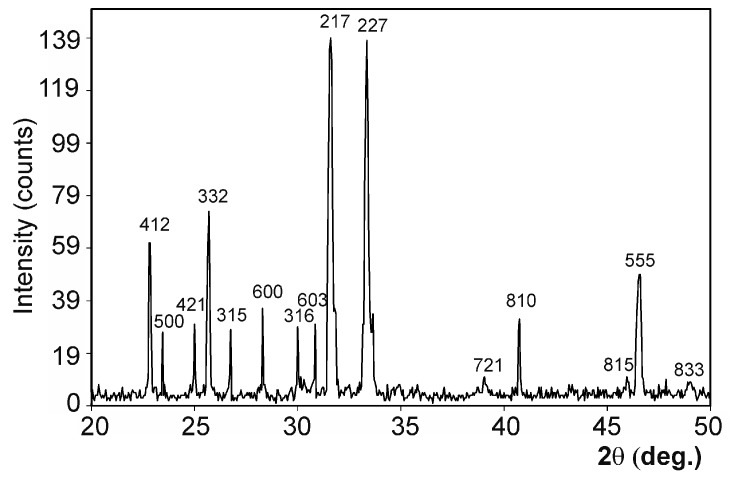
XRD pattern of the Nurse’s A powders.

**Figure 2:**
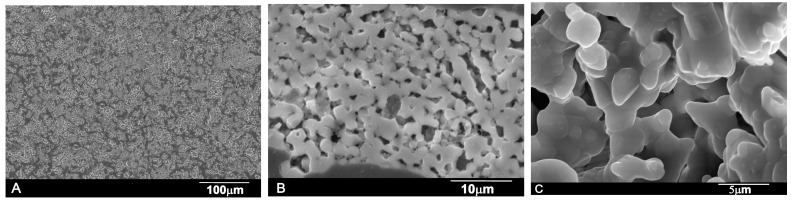
SEM images that depict (**A**,**B**) the polished Nurse’s A ceramic microstructure; (**C**) Fracture surface at a high magnification.

Figure 1 shows the XRD diffraction pattern of Nurse’s A powder ceramic. Each diffraction peak can be assigned to the characteristic reflections of 2Ca_2_SiO_4_·Ca_3_(PO_4_)_2_ (JCPDS card No. 11-0676). Figure 2A,B shows the polished surface of the obtained porous ceramic after chemical etching (0.5% acetic acid, 2 s). The figure evidenced that a monophasic material of high porosity was obtained. No critical defect was detected on any surface and a homogeneous microstructure with large spherical pores was observed at low magnification. EDS confirmed that silicon, phosphorous, and calcium were present. Figure 2C illustrates the characteristic fracture surface. The microstructure was made up of high-density aggregates with elongated pores around them. The size of the aggregates—with diameters of around 20–30 µm—allows their identification as coming from aggregates initially present in the green compacts. Such aggregates would come from milling. Fracture appears to occur by the detachment of such aggregates through the coalescence of the elongated pores. There are also cavities close to these aggregates that should be the negative of detached zones in the corresponding fracture surface. 

**Figure 3:**
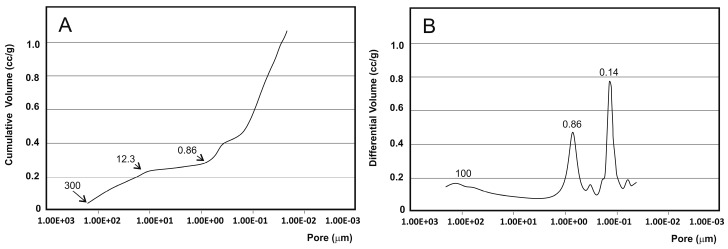
(**A**) Mercury intrusion curves of the ceramic measured by mercury porosimetry: cumulative intruded volume *vs.* pore diameter and (**B**) differential-intruded volume *vs.* pore diameter. The intrusion profiles show a small mercury penetration into pores between 300 and 12.3 µm (interparticle pores) and a significant mercury penetration into pores smaller than 0.86 µm (intraparticle pores).

When analyzing a material by mercury porosimetry, two kinds of spaces can be detected: those that correspond to the empty spaces between the particles (commonly designated by “interstices” or “interparticle” spaces) and those that correspond to the spaces of the particles themselves (known as “pores” or “intraparticle” spaces). The results obtained for porosity (Figure 3) showed that mercury penetrated to the increasingly smaller pores with increasing pressure. The cumulative curve (Figure 3A) denotes a small intrusion in pores between 300 (upper limit detection) and 12.3 µm, followed by a plateau between 12.3 and 0.86 µm where no intrusion was detected, and then a significant mercury penetration into the pores that are smaller than this value. The initial curve rise corresponded mostly to filling the interparticle spaces, whereas the later curve rise was related to the intraparticle spaces. The range of the intraparticle pores is more obvious in Figure 3B, in which two intense peaks at 0.86 and 0.14 µm are clearly visible. The smaller peak on the left (~100 µm) corresponds to the intrusion of mercury in the interparticle spaces. However, the distinction between the inter- and intraparticle spaces was not always so apparent. This interpretation aimed to elucidate the kind of information that can be extracted from the pore size distribution curves and highlights the importance of always specifying the size range of the measured pores. It should be stressed that the mercury intrusion technique is especially suited to the analysis of intraparticle pores, and is not especially suitable for measuring large spaces (300 µm).

Microstructural parameters were established to comprehensively characterize the microstructure of the material (Table 1). The obtained strength values for the material were relatively low due to the porosity of the ceramic. The results were directly related to the density of the material.

**Figure 4:**
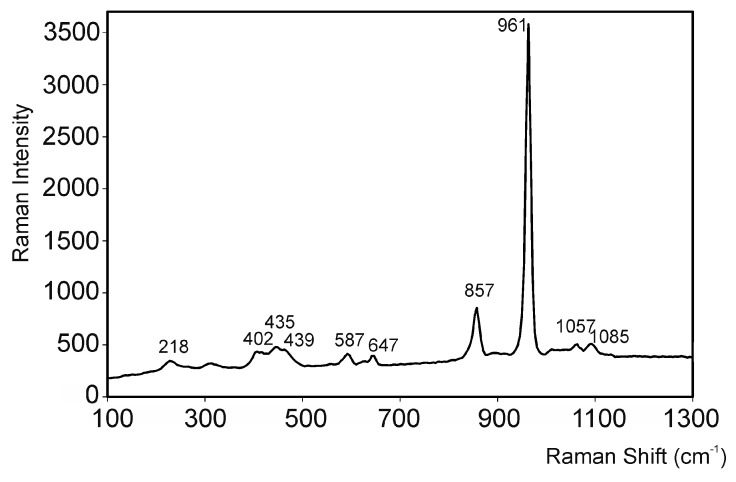
Raman spectra of Nurse’s A ceramic.

We can observe in the Raman spectra of Nurse’s A material (Figure 4) that the two most intense bands were located at 857 and 961 cm^−1^. We can also see wide and low intensity bands around 200–650, 1058, and 1084 cm^−1^. The internal modes of the SiO_4_^−4^ and PO_4_^−3^ tetrahedral units dominated the Raman spectra of crystalline silicates and phosphates. The spectra interpretation was not evident because the bands that corresponded to groups SiO_4_^4−^ and PO_4_^3−^—which formed the structure—overlapped. Given the similarities of the SiO_4_^4−^ and PO_4_^3−^ tetrahedral molecular units, both the silica calcium silicates and the hydroxyapatite crystalline phases share many similarly-spaced vibrational modes [20,21,22,23]. The main contribution of phosphate generally fell within a higher wavelength range (955–1115 cm^−1^), followed by the most intense silicate vibrations (848–879 cm^−1^), and finally within a narrower range (200–616 cm^−1^), the phosphate contribution was once again identified. 

### 2.2. Implants Characterization

**Figure 5:**
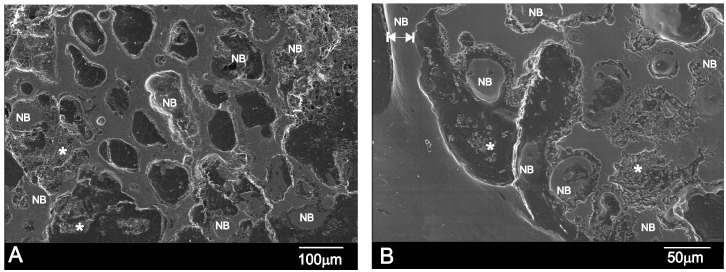
SEM images of the cross-section of Nurse’s A implant in bone after (**A**) 15 and (**B**) 30 days of implantation. Letters NB refer to the new bone tissue, while * refers to the Ca-Si-P particles as a result of the degradation process.

On day 15 post-implantation, the porous implant was well-integrated into the host tissue and formed an irregular surface boundary caused by the material’s gradual degradation (see Figure 5A). The interface between the implant and the surrounding tissue was characterized by the intermittent presence of the calcium phosphate phase and traces of silicon. In structure and morphology terms, it corresponded to new bone tissue. “NB” in Figure 5A denotes new bone, and the EDS analysis supported these findings (Table 2). In many areas, much larger-sized regions contained partly loose and exposed parent Si-Ca-P particles as a result of the material’s degradation in the physiological environment. Figure 5A denotes these particles as *. A positive effect was achieved by the formation of a much larger porous structure due to ceramic degradation as it acted as a scaffold for vascular ingrowths and osteoblast activities, which led to new bone growth inside the implanted material.

Figure 5B illustrates the massive bone colonization of the implant via the original pores in the ceramic, owing to progressive structure dissolution. As a result of these advanced processes, free material particles were found in many areas and all over the restructuring implant. An asterisk (*) and NB respectively denote the Si-Ca-P particles and ingrown bone regions in Figure 5B.

**Figure 6:**
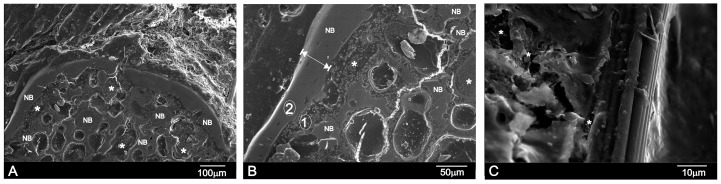
(**A**,**B**) SEM images of the cross-section of Nurse’s A implant in bone after 60 days of implantation; (**C**) A vessel present on the surface of the new bone layer in contact with the degraded Si-Ca-P material. Letters NB refer to the new bone tissue, while * refers to the Ca-Si-P particles as a result of the degradation process.

The new bone also filled the implant’s central and peripheral porosities on day 60 post-implantation (Figure 6A). The interface characterization demonstrated that the calcium phosphate phase along the implant interface periphery was well-textured since Si-Ca-P material degradation inside the implant continued. The outer product layer comprised phosphorus and calcium elements with an average Ca/P ratio of ≈2.24 (Table 3). Some cracks had readily developed close to the implant surface, and were associated with the drying process. Figure 6B offers a close-up SEM image of the 60-day implant interface. Compared with the 30-day implant (Figure 6B), the surface layer was considerably thicker on day 60. The new bone layer thickness was about 13 ± 10 μm by 15 days, which increased to about 30 ± 7 μm by 30 days, and then to about 50 ± 4 μm by day 60. Figure 6C illustrates a vessel that came into direct contact with the newly formed bone layer around the implant surface on day 60. Granular entities were identified and covered the new bone (refer to *).

According to both the EDS analysis and the high-magnification SEM images of the interface between Nurse’s A implant and natural bone, the reaction zone was composed of the Ca-P phase that contained a small quantity of Si, which had almost disappeared a short distance away from the reaction zone (Table 2). No obvious morphological differences were obtained between the newly-formed bone and the old bone into which the implants were inserted.

Table 4 provides information about the residual biomaterial and the resorption rate at different implantation times. Implant volume progressively decreased as bone formation increased along the implant periphery. Filling the central porosities of the implant led to an almost complete cortex closure on day 60. By day 60 post-implant, implantation showed extensive bone resorption (47.38% ± 1.24%). Alterations were not observed during the resorption process of the correct material, nor did any interference take place for the gradual replacement with new bone at the material’s implant site.

**Figure 7:**
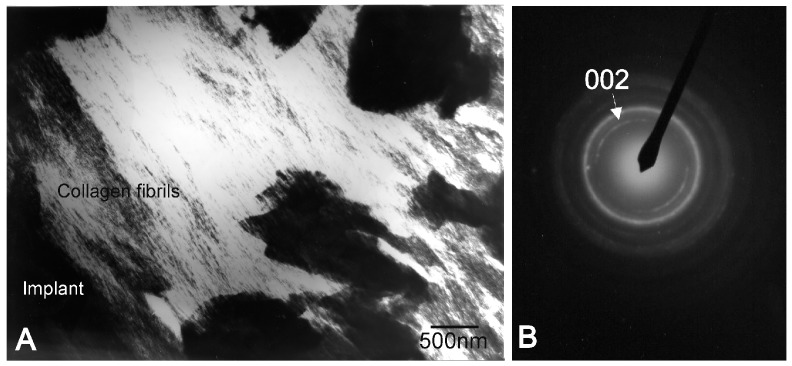
(**A**) Stained interface in a transmission electron micrograph on day 60, where well-resolved collagen fibers are observed in the newly-formed Ca-P phase; (**B**) equivalent selected area diffraction pattern of new bone tissue; thin unstained section.

The Ca-P phase was further identified by examining thin sections under a transmission electron microscope. From a structural and morphological perspective, the present research confirmed that bone was mimicked by the newly-formed phase. Figure 7 displays the implant interface and the bright field image of the stained Ca-P. The micrograph well resolved collagen fibers with the characteristic banding. The selected area diffraction pattern of the unstained thin sections also comprised the new interface phase (Figure 7B), which resulted in typical arcing in the (002) direction given the nano-apatite crystals’ preferential orientation in the collagen fiber matrix.

**Figure 8:**
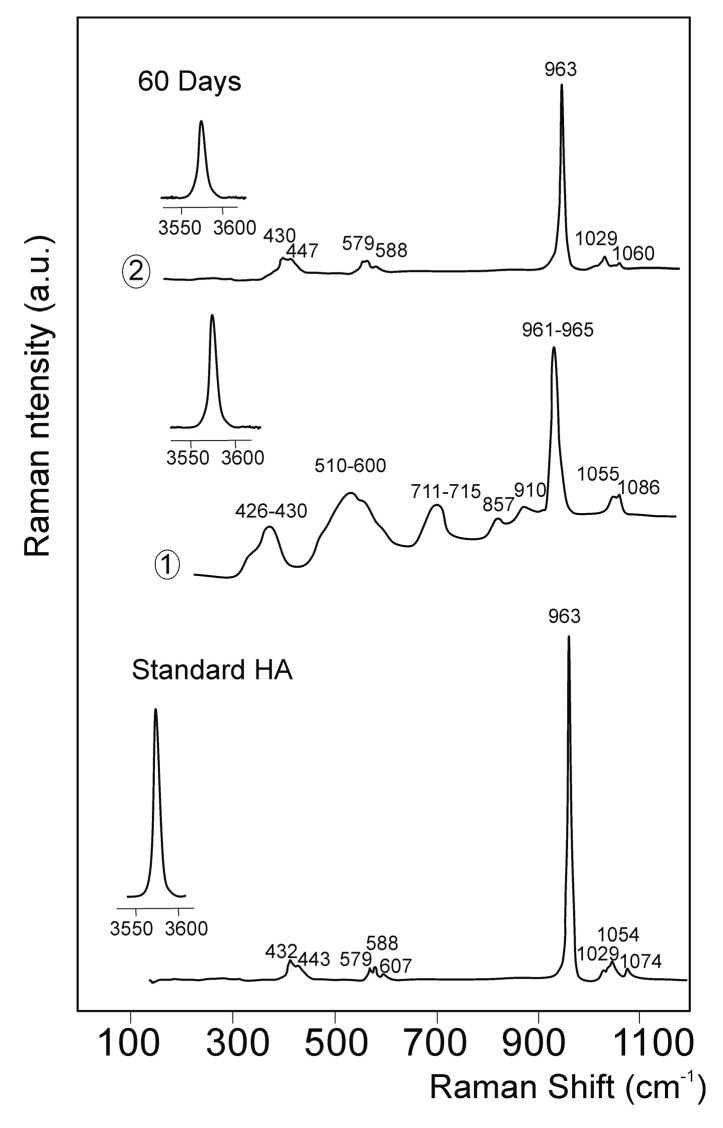
µ-Raman spectra of pure hydroxyapatite and Nurse’s A implant–bone interphase. See Figure 6B for the analyzed spots.

Figure 8 depicts the μ-Raman spectra from the reaction zone shown in Figure 6B (points 1 and 2) after 60 days of implantation in the 100–1200 cm^−1^ and 3550–3600 cm^−1^ spectral regions. To facilitate comparison, the Raman spectra of pure-Hydroxyapatite (Aldrich) was measured and is represented in Figure 8. Bands were typical of a hydroxyapatite phase [20,23], with the corresponding vibration modes of the PO_4_^3−^ group at a wavenumber of around 432 and 443 cm^−1^ (ν_2_ symmetric bending mode), 579, 588, and 607 cm^−1^ (ν_4_ antisymmetric bending mode), 963 cm^−1^ (ν_1_ symmetric stretching mode) and 1029, 1054, and 1074 cm^−1^ (ν_3_ antisymmetric stretching mode). An intense Raman peak appeared at 3576 cm^−^^1^, and was caused by the O–H stretching mode.

After 60 days of implantation, in reaction area one different bands from those of Nurse’s A (Figure 8) can be observed in the Raman spectra. The large line widths of the new bands are typical of non-crystalline materials. Bands were related to the presence of apatite and Nurse’s A amorphous phases in the material interphase. The vibrations of the ν_2_ and ν_4_ modes of the phosphate groups in the apatite phases have been reported to be around 432–450 and 580–617 cm^−1^, respectively. The presence of two broad bands at 510–600 and 711–715 cm^−1^ can be associated with the CO_3_^2−^ groups in the apatite phases [20,23], and/or with the contributions of combining the vibrations of the O–Si–O and O–P–O bonds in the amorphous-like phases [24].

Bands of around 440, 540, 590, and 700 cm^−1^ have been assigned in phosphate that contained silicate glasses in relation to the vibrations in phosphorus-oxygen units [24,25]. Bands of around 695 and 780 cm^−1^ have been reported in the Raman spectrum of calcium–phosphate glasses [24,25]. In the present work, a band at 961–965 cm^−1^ was observed close to the most intense peak of Nurse’s A ceramic (~961 cm^−1^, Figure 4) and the principal absorption peak of hydroxyapatite (963 cm^−1^, Figure 8) [20,23]. At a higher wave number, two bands were observed. The band of around 1055 cm^−1^ was most characteristic of the CO_3_^2−^ units in the structure of the carbo-apatite phases [20,23], although the vibrations related to the asymmetric stretching modes of groups Si–O–Si have also been reported in this region [21,22]. The vibrations of groups –PO_3_ and –PO_2_ in the phosphate amorphous phases could contribute to this broad band between 1000 and 1300 cm^−1^ [23,24]. An intense Raman peak appeared at 3576 cm^−1^, which was caused by the O–H stretching mode. It is noteworthy that the bands of Nurse’s A in reaction zone two disappeared, and that the intensity and crystallinity of the hydroxyapatite bands increased.

## 3. Discussion

The obvious sharp peaks and low backgrounds in Figure 1 suggest that the obtained biomaterial powders were highly crystalline. The SEM micrographs and EDS analyses showed that only one homogeneous phase was present in the obtained material. The material’s composition, established by the quantitative analysis by EDS at different sample surface points, was around 18.28% SiO_2_, 59.49% CaO, and 22.23% P_2_O_5_ (wt %), and came close to the synthesized material’s composition determined by the chemical analysis (around 18.36 wt % SiO_2_, 59.96 wt % CaO, and 21.68 wt % P_2_O_5_).

There is some controversy in the published literature as to the effect of porosity on bone regeneration. Although it has been generally recognized that large pores (>100 µm) enhance new bone formation because they allow the migration and proliferation of osteoblasts and mesenchymal cells, it has also been reported that presence of micro-porosity alters the pattern and dynamics of osteointegration [26], and might enhance ionic exchange with body fluids. It has also been reported that a nanoporous structure improves cell adhesion, proliferation, and differentiation. Nevertheless, pore interconnectivity has been indicated as a major benefit [27].

The measured porosity values (Table 1) showed intra- and inter-particle porosity, as well as total porosity. The majority of studies published in the literature present only total porosity values. However, information about pore size distribution might prove even more relevant to anticipating implant performance. A detailed analysis of the pore size distribution curves that were obtained by mercury intrusion revealed that the major contribution seems to derive from the intraparticle spaces with 15.013% of the total porosity (20.21%).

If we bear in mind the mechanism that several authors have proposed to explain the bioactive response of calcium silicate/phosphate materials [1,5,6,8,16] and the experimental results obtained herein, the reaction of Nurse’s A material *in vivo* could be described as a dissolution–transformation process. The SEM and EDS results revealed the preferential dissolution process of Nurse’s A material, while the colonization of the implant by newly formed bone on the material surface and into the pores was evidenced by SEM and TEM microscopy. The SEM observation of the cross-section of the sample implanted for 30 and 60 days proved that the process took place not only on the material’s surface, but also in the material’s internal pores. The Raman results indicated that the newly formed bone was composed of a carbohydroxyapatite phase.

This research demonstrated that Ca, P, and Si ions were released (Table 2), which favored new bone growth. High Ca and P levels could stimulate osteogenesis, given their effects on osteoblast gene expression, as Lazary *et al.* have described [28]. In normal calcified bone, Ca/P molar ratios rise with increased calcification. The presence of silicon is essential for all of these elements because it promotes mineralization processes. The porous Nurse’s A material offers biocompatibility, has good mechanical strength (Table 1), and causes no adverse inflammatory reactions at the insertion site. As it is absorbable, its rapid replacement with new bone does not react to foreign bodies, so either a slight or no inflammatory reaction took place. The same can be stated of the control samples, as their rapid replacement with new bone enabled a bone matrix within the material to be established, which conferred the receiving area similar physical properties to bone. 

There may be two reasons for the higher Ca/P ratio obtained. On the one hand, the first important factor is the release of continuous Ca from Nurse’s A ceramic. On the other hand, bone can be enriched by carbonate ions, and by producing carbonate-enriched HA. This observation suggests that the material surface could provide an optimal stratum for bone tissue ingrowth. The new bone formation mechanism can be summarized in the following steps: 

Dissolution of Nurse’s A phase with the release of Ca^2+^ and HSiO_4_^−^ as majority ions, and increased Ca^2+^, HSiO_4_^−^, OH^−^ ionic activities in the neighborhood of the reacting surface until they exceeded the solubility product of HA, led to:

Ca_7_(PO_4_)_2_(SiO_4_)_2_ + 2H_2_O → 2SiO_4_^4−^ + 2HPO_4_^2−^ + 7Ca^2+^ + 2OH^−^

This reaction started on the material’s surface, and progressed deeply into the material in the confined pores as Nurse’s A ceramic was dissolved. Apatite layer nucleation occurred through a reaction of phosphate ions with the excess calcium ions released to the medium by the ceramic. The silicate ions released by the ceramic can produce silicon HA. The SEM-EDS data indicated a very small quantity of silicon substitution in the apatite phase: 31.57/14.09/0.17 (Ca/P/Si wt %):

(6 − *x*)HPO_4_^2−^ + *y*SiO_4_^4−^ + 10Ca^2+^ + 8OH^−^ → Ca _10−*x*_ (HPO_4_)*_x_*(PO4)_6−*y*_(SiO_4_)*_y_*(OH)_2−*x*_ + 6H_2_O

The silicon that was not implicated in the reaction migrated through the medium away from the interface. Nonetheless, the diffusion of ions across the interface could stop, given the new bone layer’s thickness and structure, which was perhaps why we found Si in the new bone layer (Table 2). Bone could be enriched by carbonate ions, could produce the carbonate-enriched HA of variable CO_3_^2−^ content and lead to variable Ca/P ratios (higher than pure HA) by substituting PO_4_^3−^ by the CO_3_^2−^ groups (Table 3 and Figure 8).

What this indicates is that the new bone formation process at the interface and the new bone ingrowths into the implant would continue, provided the ion exchange mechanism between the implant and body fluids took place, assuming that the implant still remained in a biologically healthy environment. We expected this process to come to an end when the supply of Ca, P, and Si ions from the implant part to the surroundings finished. This could occur when the whole implant underwent transformation into the bone phase (and therefore its function as a hard tissue substitute material would be fulfilled), or when the diffusion of the ions across the interface stopped owing to the new bone layer’s thickness and structure. These results suggested that Nurse’s A ceramic continued to be morphologically active in the natural hard tissue environment, and was bound directly to bone through a Ca-P rich layer with a trace of Si by mimicking natural bone in terms of morphology and composition.

Our study results indicated a strong solution-mediated effect of soluble silicate ions on bone remodeling as silicate ions originated from the dissolution of Nurse’s A ceramic. So, they could play a role in accelerating the bone mineralization process around the implant. By means of electron probe microanalysis, the studies of Carlisle have stressed that silicon plays an integral role during bone mineralization processes [29]. Silicon is essential for certain biological tissues to develop and grow—e.g., bone, teeth, and some invertebrate skeletons. Previous research has shown that dietary silicon intake is positively associated with cortical mineral density, which is subject to estrogen availability in humans [11,29,30]. The present research proved that silicon was present at several concentrations in all the phases that formed on the ceramic surface during implantation (Table 2). Although the present paper emphasizes the influence of silicate ions in accelerating the apatite formation process on the surface and inside the implant, it is important to consider the cell- and solution-mediated effects of Ca-P-Si ions on the Nurse’s A ceramic osseo-integration processes, because both processes likely occurred in parallel at the implant–tissue interface.

This study also indicated that overall implant colonization was feasible because average pore size was suitable [2]. Such porosity has been reported to allow fibrovascular and bone tissue ingrowth, which have enabled direct integration with neighboring bone [31,32]. Our study also showed that the material was osteoconductive in a physiological environment. The regions with Si-Ca-P particles in the free form were identified near the new bone layer (Figure 5 and Figure 6). Granular entities were also found to cover the new bone more closely to the vessel (Figure 6C). This finding supports the “anchorage-dependence” theory, where cell proliferation, growth and differentiation require a substrate to adhere to it [31,32,33]. Osteoblasts must react with granular material before growing and proliferating on the HA surface. Granular material may contain proteins from blood, extracellular fluids, products secreted by cells, or glass particles in various stages of degradation [33,34,35,36]. When a biomaterial comes into contact with blood, a certain amount of proteins is absorbed prior to cells interacting with the material [33,34,35,36]. In particular, proteins preferentially absorb on a given material [36].

This research suggested that Nurse’s A ceramic material remained morphologically active in a naturally hard tissue environment, and was bound to bone through a Ca-P layer with traces of Si with dense bone tissue characteristics. The porous implanted material indicated that the observed gradual degradation could be attributed to its components’ different solubilities: soluble calcium, phosphate, and silicon, and suitable pore size. A large amount of newly-formed bone tissue was observed in the bone defects treated with Nurse’s A ceramic, with a resorption rate of 52.62% ± 1.12% and of 47.38% ± 1.24% for the residual biomaterial, after 60 healing days. Other studies have shown that porous HA does not significantly degrade, but remains a permanent fixture susceptible to long-term failure [4,37,38]. Although α- and β-TCP ceramics are degradable at a quicker degradation rate than HA, *in vivo* osteogenesis of sintered α- and β-TCP ceramics is far from optimal [3,5]. Recently, Mate-Sanchez *et al.* [3,5,7,39] found that Si-TCP grafts exhibited better dimensional stability and increased bone-to-implant contact with a reabsorption rate of ~71.5% for α-TCP and ~42.2%for Si-TCP after implanted *in vivo* for 60 days.

Based on these results, we believe that Nurse’s A ceramic material could be used as an alternative to natural bone because it gradually reabsorbed and also allowed new bone to grow and remodel over the whole implant volume. Future research should be conducted using standard materials, such as Si-hydroxyapatite or Si-tricalcium phosphate. The ceramic’s biological performance should be investigated in different bone defect models, such as those of critical-size or compromised situations (e.g., osteoporosis), and probably with long-term assays, as proposed in International Standard ISO-10993-5.

## 4. Materials and Methods

### 4.1. Biomaterial

Porous calcium silicophosphate ceramic was obtained by a solid-state reaction to form a stoichiometric mixture of calcium hydrogen phosphate anhydrous (CaHPO4, Panreac, Castellar del Vallès, Spain), calcium carbonate (CaCO_3_ > 99.0 wt % Fluka, St. Louis, MO, USA) with an average particle size of 13.8 µm, and silicon oxide (SiO_2_ > 99.7 wt %, Strem Chemicals, Inc., Newburyport, MA, USA) with an average particle size <50 µm. Details of this technique can be found in a previous publication [17].

The ceramic’s chemical composition was analyzed by X-ray Fluorescence (XRF, Model MagiX Super Q version 3.0 Philips, Eindhoven, The Netherlands) spectrometry. The powder material was mineralogically characterized by XRD (Bruker-AXS D8Advance, Karksruhe, Germany). A comparison was made with the database of the Joint Committee on Powered Diffraction Standards (JCPDS). 

The material’s microstructure was characterized by SEM-EDS (SEM-Hitachi S-3500N, Ibaraki, Japan) at an accelerating voltage of 20 kV. µ-Raman (Jobin Ivon T64000 spectrometer Edison, Middlesex County, NJ, USA) equipped with a microscope was used to examine the structural characteristics of the obtained ceramic. 

Information concerning the sample porosity and pore size distribution was obtained by mercury porosimetry in a Poremaster-60 GT (Quantachrome Instruments, Boyton Beach, FL, USA) within the 5.395 KPa to 410,785.062 KPa pressure range, which corresponds to a range of pore diameters between 300 and 0.0035 µm. Three samples (~0.53 g) were analyzed by this technique. A fourth sample was also used if the measured porosity values differed by more than 5%.

The particle’s real density (sample mass/Volume of the solid), excluding empty spaces, was determined by Helium gas pycnometry (Quantachrome Instruments, Boyton Beach, FL, USA). Strength was determined by the Brazilian test or the Diametric Compression of Discs Test (DCDT) [9]. The test was conducted on discs with a diameter of ~18.40 mm (D) and a thickness (t) of ~4.70 mm, (t/D ~0.25). Load was applied at a displacing rate of 0.5 mm/min of the machine frame. 

### 4.2. Animal Experimentation

#### 4.2.1. Principal Protocol

The study protocol was examined and approved by the Institutional Ethic and Animal Experimentation Committee of the University Miguel Hernandez according to Spanish Government Guidelines and European Community Guidelines for animal care (authorized No. 2014/VSC/PEA/00056 tipo2). All surgical procedures, implant insertions, and sacrifices were performed under rigorous aseptic conditions by an experienced surgeon (Dr. L. Meseguer-Olmo, authorized in small animal research). Fifteen male New Zealand rabbits were used, which weighed 3.5–4.5 kg. The ceramic was randomly implanted into critical-size defects in the tibias of these animals. 

#### 4.2.2. Surgery

**Figure 9:**
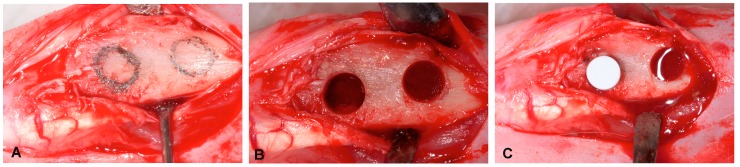
Surgical procedure. (**A**) Tibia after flap removal; (**B**) Preparation of two critical defects of 6 mm diameter located at 4 mm from the top margin with 3 mm of separation between defects; (**C**) biomaterial placement.

Two circular critical-size defects (6 mm Ø) were created in each tibia. The definition of critical size is a defect with no spontaneous closure by day 60. The surgical procedure affected the proximal-medial area of the tibias some millimeters below frontal tuberosity. Spherical surgical drills (diameter of 6 mm) operated at low rotation speed and constant irrigation to remove bone tissue (Figure 9A,B). Nurse’s A ceramic was implanted in the critical size defects created per tibia in the 15 New Zealand rabbits, which totaled 60 defects. They were divided into a ceramic-filled test group (*n* = 30) and a control group (*n* = 30) (Figure 9C).

General anesthesia included ketamine plus chlorbutol (5–8 mg/kg intravenously), 0.5–1 mg/kg acepromazine maleate as a coadjutant and 0.05 mg/kg atropine. Amoxicillin (0.1 mL/kg) was administered intramuscularly at the end of surgery.

### 4.3. Implant Characterization

Animals were sacrificed with an overdose of anaesthetics in groups of five on post-surgery days 15, 30, and 60. The specimens to undergo SEM examination were prepared from the tibia segments that contained the implant, along with surrounding tissue. Firstly, 10 vol % buffered formaldehyde solution was utilized to fix samples. Samples were then dehydrated in a series of graded alcohol solutions and were hydroxyethyl-methacrylate resin-embedded. All of the undecalcified blocks were polished with 6, 3, and 1 μm diamond pastes. After palladium-coating blocks, scanning secondary electron imaging was performed at 20 keV.

An elemental analysis was run to detect the chemical degradation process and to observe any changes in medullary composition. Thickness measurements of the Ca-P layer were taken on SEM micrographs at a high magnification. Twelve measurements per sample were taken, which gave a total of 60 measurements per implantation period.

Material resorption was assessed by comparing the SEM images before and after implantation. The Image J image analysis program (National Institutes of Health, Bethesda, MD, USA) was used to calculate the resorption rates by demarcating the area of interest as the total biomaterial upon implantation, and then measuring its perimeter and comparing it with the residual material after 15, 30, and 60 days.

The newly-formed Ca-P phase formed at the interface was morphologically and structurally identified by Transmission Electron Microscopy (TEM). Bright field images were studied with Selected Area Diffraction patterns (SAD). The Jeol Jem 2010 microscope (Jeol Ltd., Tokyo, Japan)—which operated at 200 keV and with an 80-cm camera length condition—was used for the SAD patterns. 

Raman spectroscopy was used to study the structural characteristics of the newly-formed Ca-P phase that formed at the interface. The μ-Raman microprobe instrument was equipped with a microscope and recorded the spatial resolution on the sample close to 1 μm. The 488-nm line of an Ar+ laser was used as excitation, which centered on a spot with a diameter of ~1 μm. The sample’s incident power was ~2 mW. 

### 4.4. Statistical Analysis

The statistical analysis was performed with the PASW Statistics software, v18.0.0 (SPSS Inc., Armonk, NY, USA). Values were recorded as means ± standard deviation. 

## 5. Conclusions

An *in vivo* evaluation of porous Nurse’s A ceramic was made by performing an implantation into the tibias of adult New Zealand rabbits. Despite the limitations of this animal study, we can state that Nurse’s A ceramic is a bioactive osteoconductive biocompatible material. Ceramic material favors bone regeneration at the implantation site, does not interfere with normal healing processes, and acts as an ideal matrix for new bone formation. 

The mechanism of bone bonding to the implant was the result of the dissolution and transformation of Nurse’s A implant, which brought about the formation of a new Ca-P layer at the implant interface, and the entire implanted material was transformed into a bone-like phase. From a morphological and structural viewpoint, this phase mimicked natural bone. So, we expect Nurse’s A ceramic to be satisfactorily used for repairing or substituting living bone.

## Figures and Tables

**Table 1:** Physical and mechanical properties of Nurse’s A phase.

Crystalline Size (Sherrer Å)	Shrinkage (%)	Intruded Volume (cc/g)	Total Porosity (%)	Intraparticle Porosity (%) ^a^	Interparticle Porosity (%) ^b^	Real Density (g/cm^3^)	Apparent Density (g/cm^3^)	Strength (MPa)
213	31.99 ± 0.5	0.1127	20.21	15.013	5.1969	2.13	1.72	0.60 ± 0.02

^a^ Corresponding to 0.0035 µm < pores < 1 µm; ^b^ Corresponding to 1 µm < pores < 300 µm.

**Table 2:** EDS elemental analysis of the reaction zone 15, 30, and 60 days after implantation. Mean ± SD (Median). Also, EDS elemental analysis of the Nurse’ A material and hydroxyapatite (HA) theoretical for comparative purposes.

(wt %)	O	Ca	P	Si
Nurse’ A Material				
0 day	39.21 ± 0.50 (39.21)	42.50 ± 0.50 (42.50)	9.70 ± 0.50 (9.70)	8.54 ± 0.03 (8.54)
HA Theoretical				
0 day	41.39	39.90	18.50	-
Implant				
15 days	38.9 ± 1.58 (38.9)	42.12 ± 1.66 (42.12)	10.5 ± 0.57 (10.5)	8.50 ± 1.05 (8.50)
30 days	42.10 ± 1.64 (42.10)	40.30 ± 1.54 (40.30)	9.7 ± 0.53 (9.7)	7.9 ± 0.86 (7.9)
60 days	43.60 ± 1.78 (43.60 )	39.7 ± 1.43 (39.7)	9.21 ± 0.40 (9.21)	7.5 ± 0.64 (7.5)
Interphase				
15 days	51.07 ± 1.97 (51.07)	31.46 ± 1.46 (31.46)	15.57 ± 0.96 (15.57)	1.90 ± 1.1 (1.90)
30 days	53.37 ± 1.87 (53.37)	31.27 ± 1.50 (31.27)	14.89 ± 0.87 (14.90)	0.71 ± 1.62 (0.71)
60 days	53.94 ± 1.89 (53.94)	30.94 ± 1.38 (30.94)	14.78 ± 0.84 (14.78)	0.34 ± 0.94 (0.34)
New Bone				
15 days	51.82 ± 1.96 (51.82)	32.56 ± 1.37 (32.56)	15.36 ± 0.98 (15.36)	0.26 ± 1.30 (0.26)
30 days	53.71 ± 1.88 (53.71)	31.92 ± 0.44 (31.92)	14.18 ± 0.96 (14.18)	0.19 ± 1.58 (0.19)
60 days	54.17 ± 0.91 (54.17)	31.57 ± 0.42 (31.57)	14.09 ± 0.87 (14.10)	0.17 ± 1.01 (0.17)

**Table 3:** Ca/P ratio of the reaction zone 15, 30, and 60 days after implantation. Mean ± SD (Median).

Time of Implantation	Implant	Interphase	New Bone
	Ca/P Ratio	Ca/P Ratio	Ca/P Ratio
15 days	4.01 ± 0.23 (4.01)	2.02 ± 0.54 (2.02)	2.12 ± 0.99 (2.12)
30 days	4.15 ± 0.16 (4.15)	2.10 ± 0.43 (2.10)	2.20 ± 0.67 (2.20)
60 days	4.31 ± 0.64 (4.34)	2.16 ± 0.45 (2.16)	2.24 ± 0.75 (2.24)

**Table 4:** Residual biomaterial and resorption rate 15, 30, and 60 days after implantation. Mean ± SD (Median).

Time of Implantation	Implant Material	
	Residual Biomaterial (%)	Resorption Rate
15 days	73.32 ± 2.04 (73.32)	26.68 ± 1.05 (26.68)
30 days	64.21 ± 1.07 (64.21)	35.79 ± 0.74 (35.80)
60 days	47.38 ± 1.24 (47.39)	52.62 ± 1.12 (52.62)
